# A Randomized Controlled Trial of Local Delivery of a Rho Inhibitor (VX-210) in Patients with Acute Traumatic Cervical Spinal Cord Injury

**DOI:** 10.1089/neu.2020.7096

**Published:** 2021-07-15

**Authors:** Michael G. Fehlings, Yang Chen, Bizhan Aarabi, Faiz Ahmad, Kim D. Anderson, Travis Dumont, Daryl R. Fourney, James S. Harrop, Kee D. Kim, Brian K. Kwon, Hari K. Lingam, Marco Rizzo, Ludy C. Shih, Eve C. Tsai, Alexander Vaccaro, Lisa McKerracher

**Affiliations:** ^1^ Division of Neurosurgery and Spine Program, University of Toronto and Toronto Western Hospital, Toronto, Ontario, Canada.; ^2^ Vertex Pharmaceuticals Incorporated, Boston, Massachusetts, USA.; ^3^ University of Maryland, Baltimore, Maryland, USA.; ^4^ Department of Neurosurgery, Emory University, Atlanta, Georgia, USA.; ^5^ Case Western Reserve University, Cleveland, Ohio, USA.; ^6^ Neurovascular Surgery Program, University of Arizona College of Medicine, Tucson, Arizona, USA.; ^7^ Neurosurgery, University of Saskatchewan, Saskatoon, Saskatchewan, Canada.; ^8^ Division of Spine and Peripheral Nerve Surgery, Thomas Jefferson University Hospitals, Philadelphia, Pennsylvania, USA.; ^9^ University of California Davis Health, Sacramento, California, USA.; ^10^ University of British Columbia, Vancouver, British Columbia, Canada.; ^11^ The Ottawa Hospital, Ottawa Hospital Research Institute, University of Ottawa, Ottawa, Ontario, Canada.; ^12^ Rothman Orthopaedics, Philadelphia, Pennsylvania, USA.; ^13^ BioAxone BioSciences, Inc, Boston, Massachusetts, USA.; ^14^ McGill University, Montreal, Quebec, Canada.

**Keywords:** motor recovery, randomized clinical trial, Rho inhibition, spinal cord injury, VX-210

## Abstract

Acute traumatic spinal cord injury (SCI) can result in severe, lifelong neurological deficits. After SCI, Rho activation contributes to collapse of axonal growth cones, failure of axonal regeneration, and neuronal loss. This randomized, double-blind, placebo-controlled phase 2b/3 study evaluated the efficacy and safety of Rho inhibitor VX-210 (9 mg) in patients after acute traumatic cervical SCI. The study enrolled patients 14–75 years of age with acute traumatic cervical SCIs, C4–C7 (motor level) on each side, and American Spinal Injury Association Impairment Scale (AIS) Grade A or B who had spinal decompression/stabilization surgery commencing within 72 h after injury. Patients were randomized 1:1 with stratification by age (<30 vs. ≥30 years) and AIS grade (A vs. B with sacral pinprick preservation vs. B without sacral pinprick preservation). A single dose of VX-210 or placebo in fibrin sealant was administered topically onto the dura over the site of injury during decompression/stabilization surgery. Patients were evaluated for medical, neurological, and functional changes, and serum was collected for pharmacokinetics and immunological analyses. Patients were followed up for up to 12 months after treatment. A planned interim efficacy-based futility analysis was conducted after ∼33% of patients were enrolled. The pre-defined futility stopping rule was met, and the study was therefore ended prematurely. In the final analysis, the primary efficacy end-point was not met, with no statistically significant difference in change from baseline in upper-extremity motor score at 6 months after treatment between the VX-210 (9-mg) and placebo groups. This work opens the door to further improvements in the design and conduct of clinical trials in acute SCI.

## Introduction

Acute traumatic spinal cord injury (SCI) causes severe, permanent disability attributable to loss of motor, sensory, and autonomic functions. The initial mechanical trauma sustained with SCI initiates a cascade of secondary injury mechanisms that amplify damage to the spinal cord and inhibit functional recovery.^1^ Approximately 17,000 new SCIs occur annually in the United States.^2^ There are currently no pharmacological therapies approved by the U.S. Food and Drug Administration (FDA) to augment functional recovery in persons with traumatic SCI.^3^ Because SCI impacts nearly every aspect of daily activities, even partial restoration of motor function may improve patients' autonomy and enhance quality of life.^3,4^

A major impediment to recovery after an SCI is the absence of meaningful axonal regeneration; hence, the mechanisms that govern axonal regeneration have been intensively investigated.^5^ Rho GTPases are key intracellular enzymes that regulate cytoskeletal mechanics and cellular motility.^6^ Given that Rho overactivation is a key step in the inhibition of motor neurite outgrowth and promotion of neuronal apoptosis post-SCI,^7,8^ inhibition of Rho represents a novel potential therapy. The convergence of several inhibitory signaling pathways on the Rho pathway makes it an attractive target for regenerative therapies.^3,5,7,9,10^

VX-210 is a cell-permeable derivative of a bacterial enzyme, C3 transferase, that inhibits Rho activity through covalent modification and therefore can potentially block Rho-mediated axonal growth cone collapse and inhibit neuronal apoptosis post-SCI.^3,7,11^ Pre-clinical studies indicate that VX-210 has neuroregenerative and -protective effects and can promote functional recovery post-SCI. In a mouse model of acute SCI, VX-210 applied extradurally at the time of injury or delayed by 24 h significantly improved open-field locomotion compared to controls at 16 days post-injury.^12^

In an earlier open-label, multi-center phase 1/2a dose-ranging study, the results of treatment with VX-210 (at the time, BA-210) were encouraging based on American Spinal Injury Association (ASIA) motor scale scores in patients with cervical SCI. Safety and tolerability were demonstrated with the doses studied (range, 0.3–9.0 mg), and adverse events (AEs) were typical of those observed with acute SCI. The largest changes in ASIA motor scores were noted in the cervical injury cohorts treated with 1 and 3 mg of VX-210 (average improvement of 21.3 and 27.3 points, respectively, at 12 months). Whereas there was little effect in the thoracic injury cohort, an overall change was observed in patients with cervical SCI. The motor recovery observed suggested that VX-210 may increase neurological recovery after complete SCI.^5^

Therefore, we initiated the phase 2b/3 study described here (NCT02669849) to test the hypothesis that VX-210 applied to the dura overlying the injured spinal cord would augment motor recovery after acute cervical SCI, with the potential for improvement in quality of life of persons living with SCI.^3^

## Methods

### Participants and study design

**FIG. 1.**
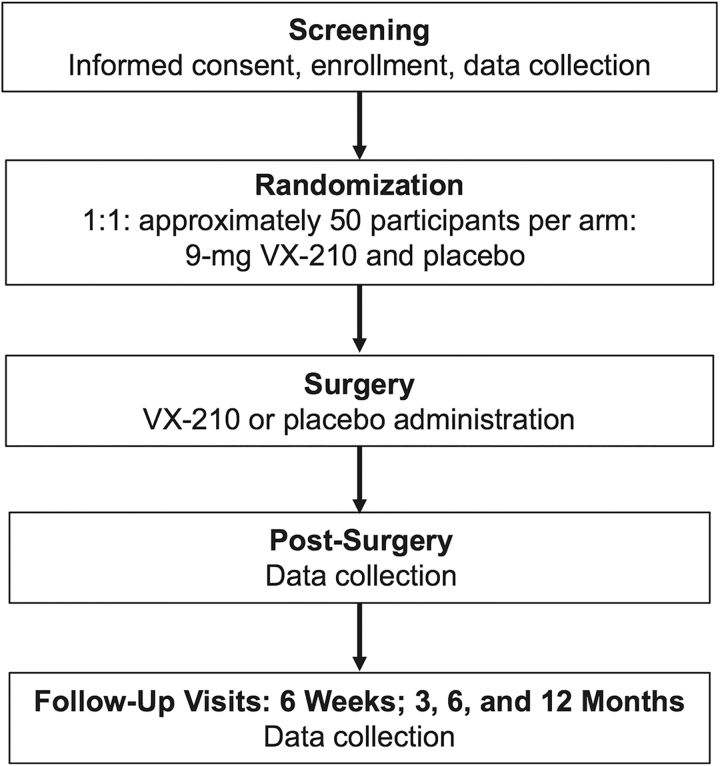
Study design. The VX-210 (3-mg) treatment arm is not reflected in this schematic.

This phase 2b/3, randomized, double-blind, placebo-controlled, multi-center study examined the efficacy and safety of VX-210 treatment (Fig. 1). Participants were enrolled at 28 sites in the United States and five sites in Canada. The study included male and female patients, 14–75 years of age (inclusive), with acute traumatic SCIs, C4–C7 (motor level) on each side, and ASIA Impairment Scale (AIS) Grade A or B. Patients with AIS Grade A and a C4 motor level on each side were required to have ≥1 point of motor activity between C5 and T1 on ≥1 side; patients with AIS Grade B and a C4 motor level on each side were required to have ≥1 point of motor activity between C5 and C7 on ≥1 side.^3^ Eligible patients had a pre-operative computed tomography (CT) and/or magnetic resonance imaging (MRI) scan and were scheduled to undergo spinal cord decompression/stabilization surgery within 72 h after injury. Patients with a body mass index ≥40 kg/m^2^, who were pregnant or breastfeeding, or who had an acute SCI from a gunshot or penetrating/stab wound, non-traumatic SCI, brachial plexus injury, complete spinal cord transection, or multi-focal SCI were excluded. Additional key exclusion criteria included:

- ≥1 upper-extremity muscle group untestable during International Standards for Neurological Classification of Spinal Cord Injury (ISNCSCI) screening examination;
- Altered mental capacity precluding reliable ISNCSCI examination;
- Inability to undergo decompression/stabilization surgery within 72 h after injury;
- Participation in another clinical study for acute SCI without sponsor approval;
- Known immunodeficiency, including human immunodeficiency virus or use of immunosuppressive or cancer chemotherapeutic drugs;
- History of an adverse reaction to a fibrin sealant or its components; and
- Any significant medical or psychiatric comorbidities that significantly interfere with study enrollment, outcomes, or assessments, as judged by the investigator.

Patients were randomized to receive a single 9-mg dose of VX-210 in fibrin sealant or placebo (buffer solution) in fibrin sealant at a 1:1 ratio with goal enrollment of ∼100 patients. Assuming that patients in the 9-mg VX-210 group had a 4-point greater improvement in upper-extremity motor score (UEMS) than patients in the placebo group, with a standard deviation (SD) of 6.0, ∼50 patients per group would provide ≥80% power to detect a statistically significant treatment effect at 6 months. Anticipated withdrawal of 10% of patients before the 6-week follow-up assessment was also accounted for in the sample-size calculation. Randomization was stratified by age (<30 vs. ≥30 years of age) and AIS grade (A vs. B with sacral pinprick preservation vs. B without sacral pinprick preservation). A third treatment group receiving 3 mg of VX-210 was initially included in the protocol, but was subsequently removed in April 2017 through a protocol amendment to accelerate completion of the trial and maximize target engagement. The higher dose, 9 mg, was chosen to maximize target engagement because the patient numbers in the cervical SCI cohort of the previous study were too small for a true dose-response relationship to be assessed.^5^

Patients were followed up for up to 12 months after treatment. At specified time points, patients were evaluated for medical, neurological, and functional changes, and serum was collected for pharmacokinetics (PK) and immunological analyses. An interim futility analysis was conducted when ∼33% of enrolled patients had completed the 6-month follow-up visit. The study was planned to be stopped for futility if conditional power^13^ was <10.6% or the observed primary end-point treatment effect size was <1.19. The study was ended prematurely because of low conditional power after the pre-defined efficacy-based interim futility analysis was completed. Patients who were prematurely terminated from the study were required to complete an early termination visit, at which efficacy and safety assessments were performed. If a specific study visit (e.g., 6-month follow-up) was the closest to the early termination visit among all the study visits, then the assessments from the early termination visit were assigned to the corresponding study visit for analysis purposes and used in the final analysis, which was conducted after study closure.

### Dose and drug delivery

VX-210 was administered topically in a fibrin sealant to the dura mater (extradural surface) of the spinal cord. The one-time dose of VX-210 or placebo was administered by a surgeon directly to the dura mater of the spinal cord at the site of injury during decompression/stabilization surgery that commenced within 72 h after SCI.

### Outcome measures

The primary efficacy end-point was the change in UEMS from baseline at 6 months after treatment. Secondary objectives included assessments of sensation and motor activity, as well as improvements in daily function based on analyses of the activities of daily living and requirements for attendant care. Secondary end-points included the following:

- Spinal Cord Independence Measure (SCIM) III self-care subscore at 6 months^14^;
- Capabilities of Upper-Extremity Test (CUE-T) score at 6 months. CUE-T is an evaluation of a patient's ability to perform specific functional movements or tasks with the arms and hands^15^;
- Graded Redefined Assessment of Strength, Sensibility, and Prehension (GRASSP) quantitative prehension score at 6 months. GRASSP is an assessment of a patient's ability to perform specific functions with the arms, hands, and fingers^16^;
- AIS grade conversion from baseline at 6 months;
- Motor-level change from baseline at 6 months; and
- PK parameters of VX-210.

### Safety and adverse events

Safety evaluations included AEs, vital signs, electrocardiograms (ECGs), clinical laboratory tests (i.e., serum chemistry, hematology, coagulation, and urinalysis), physical examinations, surgical-site examinations, and immunogenicity measures. Safety and tolerability data were reviewed by an independent data monitoring committee to ensure the safety of enrolled patients.

### Statistical analysis

Statistical analysis was performed using SAS^®^ Software (Version 9.4; SAS Institute Inc., Cary, NC). Categorical variables were summarized using counts and percentages, and continuous variables were summarized by means and SDs. Descriptive summaries of demographics and baseline characteristics, primary efficacy analysis, and secondary efficacy analysis were performed for randomized patients who received the study drug or placebo. Descriptive summaries for safety were performed for all patients who received the study drug or placebo. The primary efficacy analysis was based on a mixed-effects model for repeated measures (MMRM). The model included the change from baseline in UEMS at 6 weeks, 3 months, 6 months, and 12 months after treatment as the dependent variable; treatment, visit, and treatment-by-visit interaction as fixed effects; and subject as a random effect, with adjustment for age and AIS grade at baseline. With use of the MMRM and the assumption that unobserved values are missing at random, no missing data were explicitly imputed. Secondary efficacy end-points were summarized descriptively. The 3-mg VX-210 treatment group was not used in the statistical modeling, but was included in the safety reporting.

### Ethics

The study was conducted in accordance with the International Conference on Harmonization Guideline for Good Clinical Practice, the ethical principles in the Declaration of Helsinki, and local laws and regulations. An institutional review board or independent ethics committee provided approval for each participating site. All participants (or their legally appointed and authorized representative) provided informed consent, and assent if applicable.

## Results

### Patient disposition and baseline characteristics

**FIG. 2.**
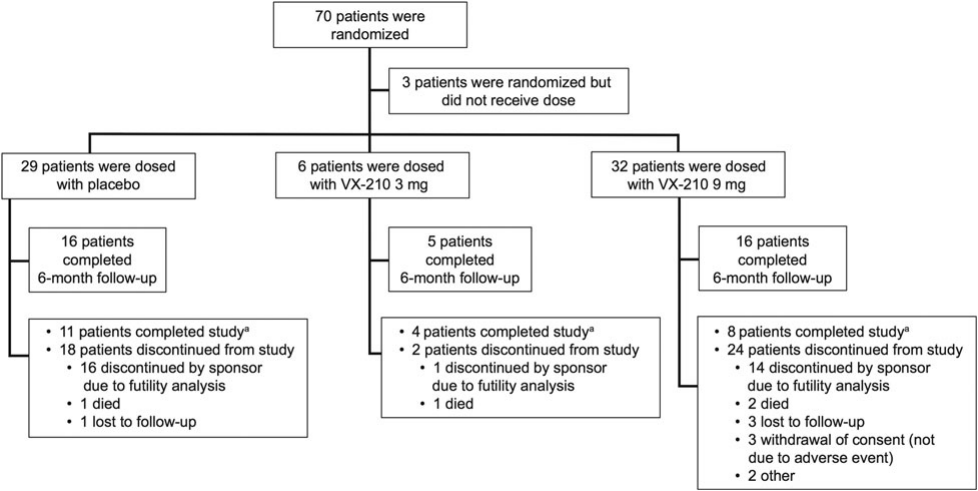
Patient disposition. ^a^Completed 12-month follow-up visit.

A total of 70 patients were randomized (Fig. 2), and 67 received study drug or placebo before the study was terminated; 37 patients completed the 6-month follow up. Patient demographics and baseline characteristics are summarized in Table 1. Twenty-four of 32 patients (75%) in the VX-210 (9-mg) group were categorized as AIS Grade A at baseline versus 20 of 29 patients (69%) in the placebo group. Motor levels were balanced across the VX-210 (9-mg) and placebo groups at baseline, with the majority of patients in both groups having injuries at the C5 level on each side (Supplementary Table S1). Twelve of 32 patients (37.5%) in the VX-210 (9-mg) group were <30 years of age at baseline compared to 8 of 29 patients (27.6%) in the placebo group.

**Table 1.** Demographics and Baseline Characteristics^a^

Characteristic	Placebo*N* = 29	VX-210**(3 mg)*N* = 6	VX-210**(9 mg)*N* = 32	Total* N* = 67
Male, *n* (%)	21 (72.4)	4 (66.7)	26 (81.3)	51 (76.1)
Age, mean (SD), years	43.8 (17.4)	39.5 (24.4)	43.4 (17.3)	43.2 (17.8)
Race, *n* (%)				
White	19 (65.5)	3 (50.0)	21 (65.6)	43 (64.2)
Black or African American	6 (20.7)	0	5 (15.6)	11 (16.4)
Asian	2 (6.9)	0	3 (9.4)	5 (7.5)
American Indian or Alaska Native	1 (3.4)	0	2 (6.3)	3 (4.5)
Not collected per local regulations	0	2 (33.3)	0	2 (3.0)
Other	1 (3.4)	1 (16.7)	1 (3.1)	3 (4.5)
Baseline AIS grade, *n* (%)				
A	20 (69.0)	4 (66.7)	24 (75.0)	48 (71.6)
B	9 (31.0)	2 (33.3)	6 (18.8)	17 (25.4)
With sacral pinprick preservation	6 (66.7)	2 (100.0)	5 (83.3)	13 (76.5)
Without sacral pinprick preservation	3 (33.3)	0	1 (16.7)	4 (23.5)
C	0	0	1 (3.1)	1 (1.5)
Baseline UEMS score, mean (SD)	13.00 (8.96)	10.17 (6.59)	14.03 (8.33)	13.24 (8.43)
Medical history, *n* (%)^b^				
Hypertension	9 (31.0)	1 (16.7)	10 (31.3)	20 (29.9)
Hypotension	6 (20.7)	2 (33.3)	6 (18.8)	14 (20.9)
Anxiety	7 (24.1)	1 (16.7)	4 (12.5)	12 (17.9)
Bradycardia	5 (17.2)	1 (16.7)	5 (15.6)	11 (16.4)
Respiratory failure	4 (13.8)	3 (50.0)	4 (12.5)	11 (16.4)
Skin laceration	5 (17.2)	1 (16.7)	5 (15.6)	11 (16.4)

*n* indicates number of patients with non-missing assessments at baseline for each variable or category; *N* indicates number of patients randomized and dosed.

^a^ Percentage for “with sacral” and “without sacral” is calculated using number of patients with Grade B as the denominator. All other percentages are based on the number of patients randomized and dosed. Medical conditions were coded using Medical Dictionary for Regulatory Activities Version 21.1. A patient with multiple conditions within a category was counted only once within that category.

^b^ Medical history for conditions occurring in >15% of total patients.

AIS, American Spinal Injury Association Impairment Scale; SD, standard deviation; UEMS, upper extremity motor score.

### Pre-specified efficacy analyses

The change from baseline in UEMS at each visit is summarized in Table 2. According to the MMRM, the primary efficacy end-point was not met, with least squares means (standard error) of change from baseline in UEMS at 6 months of 10.11 (1.97) and 10.80 (1.73) points for the VX-210 (9-mg) and placebo groups, respectively (least squares mean treatment difference between the VX-210 [9-mg] vs. placebo groups, −0.69 [95% confidence interval, −5.08 to 3.69]; *p* = 0.7519). The results of the secondary efficacy analysis support the conclusion of the primary efficacy analysis and are summarized in Table 3.

**Table 2.** Summary of Change from Baseline in UEMS at Each Visit

Change from baseline to each follow-up	Statistic	Placebo	VX-210 (9 mg)
		N = 29	N = 32
6-week follow-up	*n*	25	27
	Mean (SD)	4.16 (6.91)	3.96 (7.25)
3-month follow-up	*n*	24	22
	Mean (SD)	6.42 (7.79)	6.91 (7.48)
6-month follow-up	*n*	20	16
	Mean (SD)	8.90 (9.48)	8.69 (7.35)
12-month follow-up	*n*	13	22
	Mean (SD)	13.23 (9.91)	8.67 (9.10)

*n* indicates number of patients with non-missing assessments at the corresponding visit; *N* indicates number of patients randomized and dosed.

SD, standard deviation; UEMS, upper extremity motor score.

**Table 3.** Summary Statistics for Secondary Efficacy End-Points

End-point at 6 months after treatment	Statistic	Placebo*N* = 29	VX-210 (9 mg)*N* = 32
SCIM III Self-Care Subscore	*n*	19	17
	Mean (SD)	6.2 (7.0)	5.9 (6.6)
CUE-T Score	*n*	15	15
	Mean (SD)	39.6 (38.4)	42.8 (41.1)
GRASSP Quantitative Prehension Score	*n*	19	18
	Mean (SD)	18.9 (19.4)	20.1 (21.6)
AIS grade responders, *n* (%)^a^	*n*	20	15
	Yes	6 (30.0)	4 (26.7)
	No	14 (70.0)	11 (73.3)
Motor level responders, *n* (%)^b^	*n*	20	15
	Yes	7 (35.0)	5 (33.3)
	No	13 (65.0)	10 (66.7)

*n* indicates number of patients with non-missing assessments at the corresponding visit; *N* indicates number of patients randomized and dosed.

^a^ An AIS responder was defined as a patient with improvement by ≥2 AIS grades (i.e., baseline AIS Grade A changed to Grade C, D, or E; baseline AIS Grade B changed to D or E at 6 months after treatment).

^b^ A motor-level responder was defined as a patient with improvement by ≥2 motor levels on either side of the body (i.e., baseline level C4 changed to C6, C7, or C8 on the left; or baseline level C5 changed to C7 or C8 on the right, etc.).

AIS, American Spinal Injury Association Impairment Scale; CUE-T, Capabilities of Upper Extremity Test; GRASSP, Graded Redefined Assessment of Strength, Sensibility and Prehension; SCIM, Spinal Cord Independence Measure; SD, standard deviation.

PK revealed leakage of drug into the blood after the extradural application. Modeling was not conducted because of early termination of the study. Twenty-six patients in the VX-210 (9-mg) group had analyzable PK data, and in these patients, peak concentration time varied from 3 (*n* = 15 [58%]) to 6 (*n* = 5 [19%]) to 12 h (*n* = 5 [19%]) and, in 1 patient, 24 h (*n* = 1 [4%]); median peak concentrations were 1.70, 3.71, 6.44, and 4.34 ng/mL, respectively, with concentrations ranging from 0.32 to 60.60 ng/mL. All 6 patients in the VX-210 (3-mg) group had analyzable PK data, and peak concentration time varied from 6 (*n* = 3 [50%]) to 12 (*n* = 1 [17%]) or 24 h (*n* = 2 [33%]); median peak concentrations were 0.47, 1.78, and 0.89 ng/mL, respectively, with a range from 0.29 to 4.51 ng/mL.

### Safety

A single dose of VX-210 was generally well tolerated, and the AE profile was typical for persons with cervical SCI (Table 4). All patients had ≥1 AE and these were considered mild (*n* = 5 [7.5%]), moderate (*n* = 25 [37.3%]), severe (*n* = 20 [29.9%]), or life-threatening (*n* = 17 [25.4%]) in severity. The majority of patients had AEs that were considered unrelated (*n* = 50 [74.6%]) or unlikely related (*n* = 14 [20.9%]) to the investigational drug. Three patients (VX-210 [9 mg], *n* = 2; placebo, *n* = 1) had AEs considered possibly related to study drug: In the VX-210 (9-mg) group, 1 patient had increased hepatic enzyme and 1 patient developed a seroma at the surgical site associated with cord compression; in the placebo group, 1 patient had increased aspartate aminotransferase.

**Table 4.** Overview of Adverse Events^a^

Category	Placebo	VX-210 (3 mg)	VX-210 (9 mg)	Total
	N = 29	N = 6	N = 32	N = 67
	n (%)	n (%)	n (%)	n (%)
Patients with any AEs	29 (100.0)	6 (100.0)	32 (100.0)	67 (100.0)
Patients with AEs by maximum severity				
Mild	2 (6.9)	0	3 (9.4)	5 (7.5)
Moderate	11 (37.9)	0	14 (43.8)	25 (37.3)
Severe	8 (27.6)	4 (66.7)	8 (25.0)	20 (29.9)
Life-threatening	8 (27.6)	2 (33.3)	7 (21.9)	17 (25.4)
Patients with SAEs	18 (62.1)	6 (100.0)	16 (50.0)	40 (59.7)
Patients with AEs leading to death	0^b^	1 (16.7)	2 (6.3)	3 (4.5)^b^
Most common AEs^c^				
Urinary tract infection	20 (69.0)	4 (66.7)	20 (62.5)	44 (65.7)
Pyrexia	13 (44.8)	3 (50.0)	17 (53.1)	33 (49.3)
Decubitus ulcer	11 (37.9)	4 (66.7)	15 (46.9)	30 (44.8)
Pneumonia	9 (31.0)	5 (83.3)	15 (46.9)	29 (43.3)
Muscle spasms	9 (31.0)	3 (50.0)	14 (43.8)	26 (38.8)
Insomnia	11 (37.9)	2 (33.3)	9 (28.1)	22 (32.8)
Hypotension	10 (34.5)	0	11 (34.4)	21 (31.3)
Neuralgia	7 (24.1)	2 (33.3)	10 (31.3)	19 (28.4)
Anemia	8 (27.6)	2 (33.3)	6 (18.8)	16 (23.9)
Anxiety	6 (20.7)	1 (16.7)	9 (28.1)	16 (23.9)
Nausea	7 (24.1)	3 (50.0)	5 (15.6)	15 (22.4)
Depression	7 (24.1)	1 (16.7)	6 (18.8)	14 (20.9)
Neurogenic bladder	5 (17.2)	0	8 (25.0)	13 (19.4)
Bradycardia	7 (24.1)	1 (16.7)	4 (12.5)	12 (17.9)
Constipation	4 (13.8)	0	7 (21.9)	11 (16.4)
Musculoskeletal pain	5 (17.2)	1 (16.7)	5 (15.6)	11 (16.4)

n indicates number of patients with AEs in the corresponding category; *N* indicates number of patients dosed.

^a^ AE data were coded using Medical Dictionary for Regulatory Activities Version 21.1. Most common AEs are listed by preferred term in descending order of total. A patient with multiple events within a category was counted only once within that category.

^b^ One patient in the placebo group died during the study (unknown cause, 9 months after the patient received placebo). The event occurred outside the protocol-defined treatment-emergent period (28 days after treatment/the last available visit) for patients who do not complete the follow-up visits, as was the case with this patient.

^c^ AEs occurring in ≥15% of total patient population.

AE, adverse event; SAE, serious adverse event.

Forty patients (VX-210 [3 mg], 6 [100%]; VX-210 [9 mg], 16 [50%]; placebo, 18 [62.1%]) had serious AEs. Of these, 3 patients died: 1 patient [16.7%] in the VX-210 (3-mg) group (respiratory and renal failure) and 2 patients [6.3%] in the VX-210 (9-mg) group (respiratory complications). In addition, 1 patient in the placebo group died (unknown cause) after the treatment-emergent period. The deaths were considered unrelated to the investigational drug. There were no clinically relevant trends attributable to treatment with VX-210 identified from laboratory measurements, vital signs, physical examinations, surgical-site examinations, or ECG results.

## Discussion

This study represents the first randomized, controlled trial evaluating a targeted approach to overcome inhibitory factors in the central nervous system to promote repair, plasticity, and regeneration. The study was prematurely ended because of the pre-defined efficacy-based interim futility analysis. The primary efficacy end-point was not met, with no statistically significant difference in change from baseline in UEMS at 6 months after treatment observed between the VX-210 (9-mg) and placebo groups. Analyses of the secondary efficacy end-points also did not meet significance. The final analysis confirmed the conclusions drawn from the interim analysis. The decision to terminate the study prematurely was based only on the efficacy-based futility boundary and not on any identified safety issues. The trial included a significantly larger number of treated patients and treating sites than the phase 1/2a study. The failure to replicate the motor improvement found in the preceding study (in which in the cervical cohort, the overall average motor-score improvement from baseline was 18.6 points)^5^ may suggest that heterogeneity of patients, rehabilitation within the first 6 months of injury, drug delivery, or a combination of these factors may have played a role.

With a small number of patients with AIS Grade B (Table 1), analysis for presence of sacral-segment pinprick preservation was not helpful. The large variability in the CUE-T and GRASSP scores can shed light on the design of future studies using these two end-points and suggests that enhanced training in these outcomes instruments or refining use to subcomponents of the tests may be required.

The protocol was amended during the study because of difficulties in patient recruitment. The 9-mg group was chosen to maximize target engagement despite trends reported in the phase 1/2a study of VX-210^5^; the phase 1/2a study had small patient numbers in all dose groups, with only 2 patients in the cervical SCI 9-mg group.^5^ In the current study, 6 patients received the 3-mg dose before the protocol was modified to remove this treatment arm. Two patients who were deemed AIS Grade B at baseline and received the 3-mg dose were determined to be AIS grade responders at 6 months after treatment (i.e., improved ≥2 grade levels from baseline). Of 4 patients who were deemed AIS Grade A at baseline and received the 3-mg dose, 2 had 6-month AIS grade results available, one of whom showed a ≥2-level improvement from baseline. The VX-210 (3-mg) group was not included in the efficacy analysis because of the protocol change and low sample size. The sample size of the patients receiving the 3-mg dose (*n* = 6) is insufficient to draw conclusions about the potential impact of this dose.

Although there are currently no FDA-approved pharmacological therapies to augment motor function and functional recovery in persons with traumatic SCI,^3^ recruitment of patients for neurotherapeutic clinical trials can be a challenge. The most fundamental limitation on recruitment lies in the demographics of SCI, including the number of persons injured, and the proportion of the population with access to a trial center within the window available for treatment. Many of the details of study design further restrict the eligible population, which is smaller in the acute population than the chronic population.^17^ The efforts in identifying, consenting, and enrolling patients in the current study, however, demonstrate that such studies are feasible. In conducting this trial, we learned the importance of providing 24/7 on-call support staffed by research personnel.

The method of drug delivery and optimal dosing are important considerations when considering the outcomes of this study.^17^ Extradural delivery at the time of surgery minimizes damage to the spinal cord and limits systemic exposure by restricting delivery of VX-210 to the injury area.^12^ Consistent with findings in the first clinical study,^5^ PK results indicate that the 9-mg dose resulted in higher blood levels of VX-210 than the 3-mg dose. A potential downside of extradural delivery relates to variability in penetration through the dura and cerebrospinal fluid, and into the injured spinal cord. Direct injection into the spinal cord parenchyma was felt to be clinically unacceptable in acutely injured patients because of the potential for further injury.^12,18^ Hence, there is a need to optimize local delivery strategies through the use of biomaterial-based delivery methods—for example, to enhance penetration of the injured spinal cord.^18,19^ Additional pre-clinical studies, using larger animal models of SCI with thicker dura and a larger cerebrospinal fluid compartment surrounding the spinal cord, could inform such delivery optimization studies.

The rate of futility is high in clinical studies of acute traumatic SCI, and substantial need remains for effective treatment options with favorable risk-benefit ratios. Despite this clinical study being discontinued for futility, Rho remains a target of interest in acute traumatic SCI, and lessons from this trial may assist in the development of effective treatments in future studies. Phase 3 clinical study experience with aducanumab, a recombinant IgG1 antibody under FDA review for the treatment of prodromal Alzheimer's disease, underscores the challenges of interpreting negative data in trials stopped for futility.^20,21^

Clinical study design for future clinical trials should be optimized. The cumulative burden of follow-up measures may affect the willingness of potential patients to commit to a study and may have contributed to declines in participation in our current study.^17^ Potential solutions could include the use of telehealth-based technologies to conduct outcomes assessments.^22^ Imaging evaluation is part of the recognized standard of care for patients with traumatic SCI.^3^ Although CT and/or MRI were essential diagnostic tools included in this study, they do not yet possess the resolution to be used in recovery follow-up or outcome prediction.^5^ Novel quantitative MRI techniques facilitate improved assessment of microstructural changes induced by injury in the spinal cord and may show applicability for future studies.^23,24^ Of note, work in this field is showing promise, and it is anticipated that MRI may become a useful biomarker for SCI outcomes.^24^ In future clinical studies, it may also be beneficial to centralize collection and analysis of imaging data.

## Conclusion

Overall, VX-210 was generally well tolerated in patients with acute traumatic SCI but failed to meet the primary efficacy end-point at a pre-defined interim futility analysis. Successful execution of the current study demonstrates the feasibility of hypothesis testing of therapeutic intervention in acute cervical SCI. Future studies should enhance methods for local delivery of pharmacological agents to the injury site, examine improved strategies to predict the heterogeneity of patient recovery after cervical SCI, and focus on strategies to enhance enrollment and retention of patients.

## Supplementary Material

Supplemental data

## Data Sharing Statement

Vertex is committed to advancing medical science and improving patient health. This includes the responsible sharing of clinical trial data with qualified researchers. Proposals for the use of these data will be reviewed by a scientific board. Approvals are at the discretion of Vertex and will be dependent on the nature of the request, the merit of the research proposed, and the intended use of the data. Please contact CTDS@vrtx.com if you would like to submit a proposal or need more information.

## Supplementary Material

VX15-210-101 Study Group
