# Supplementary material for: A Randomized Controlled Trial of Local Delivery of a Rho Inhibitor (VX-210) in Patients with Acute Traumatic Cervical Spinal Cord Injury
Source: J Neurotrauma. 2021 Jul 15;38(15):2065–72. doi: 10.1089/neu.2020.7096 (PMC8309435; doi:10.1089/neu.2020.7096)
Supplement: Supplemental data [file Supp_Data.docx]

**Supplementary Material**

**Investigators and sites**

The VX15-210-101 study group included Michael Fehlings, Toronto Western Hospital, University Health Network; Gregory Hawryluk, Clinical Neurosciences Center, University of Utah Health; Faiz Ahmad, Grady Memorial Hospital; Jean-Marc Mac-Thiong, Hopital du Sacre-Coeur de Montreal; Alexander Vaccaro, Rothman Institute; Philippe Mercier, St. Louis University, Department of Internal Medicine; Jefferson Wilson, St. Michael's Hospital; Eve Tsai, The Ottawa Hospital; Patrick Hitchon, The University of Iowa Hospitals and Clinics; James Wilberger, Allegheny General Hospital; Travis Dumont, University of Arizona Clinical and Translational Science (CATS) Research Center; Paul Arnold, University of Kansas Medical Center; Bizhan Aarabi, University of Maryland; Ann Parr, University of Minnesota; Wilson Ray, Washington University School of Medicine/St. Louis Children's Hospital; Bradley Jacobs, University of Calgary, Department of Clinical Neurosciences, Foothills Medical Centre; Eric Marvin, Carilion Roanoke Memorial Hospital; Albert Yee, Sunnybrook Health Sciences Centre; David Okonkwo, University of Pittsburgh Medical Center Health System; John Hamilton, Inova Fairfax Medical Campus; Yi Lu, Brigham & Women's Hospital; Jonathan Grauer, Yale New Haven Hospital; Sean Christie, Queen Elizabeth II Health Sciences Center; Efstathios Papavassiliou, Beth Israel Deaconess Medical Center; Paul Park, Michigan Medicine; Arthur Jenkins, Icahn School of Medicine at Mount Sinai; Kee Kim, University of California Davis Medical Center; Steven Yocom, Cooper University Hospital; Patrick Hsieh, University of Southern California; Jerome Paquet, Institut Universitaire de Readaptation; Daryl Fourney, University of Saskatchewan; George Cybulski, Northwestern University Medical School; Brionn Tonkin, Hennepin County Medical Center; Francis Farhadi, Ohio State University; John Kelleher, Penn State Milton S. Hershey Medical Center; Patrick McCormick, ProMedica Toledo Hospital/Toledo Children's Hospital/Pediatric Pulmonary & Cystic Fibrosis Center; Jens Chapman, Swedish Health Services; Jonathan Miller, Rainbow Babies and Children's Hospital/University Hospitals Cleveland Medical Center; Fernando Vale, University of South Florida Department of Neurosurgery and Brain Repair; James Lindley, Memorial Health University Medical Center; Gazanfar Rahmathulla, University of Florida, Jacksonville; Jefferson Chen, UC Irvine Medical Center; Louis Harkey, University of Mississippi Medical Center; Thomas Altstadt, Kosair Charities Pediatric Clinical Research Unit; John Shin, Massachusetts General Hospital; Brian Kwon, Vancouver Hospital and Health Sciences Centre; Roxie Albrecht, OU Medical Center Hospital; Derek Taggard, University of California, San Francisco-Fresno; Adetokunbo Oyelese, Brown University/Rhode Island Hospital; Viktor Bartanusz, University of Texas Health Science Center at San Antonio; James Harrop, Thomas Jefferson University Hospital; Rajiv Saigal, Harborview Medical Center; Eeric Truumees, Dell Seton Medical Center at The University of Texas; Patrick Pritchard, University of Alabama at Birmingham Hospital; Jerry Lewis, University of New Mexico Hospital (UNMH); Brian Cameron, VCU Health; Rita Hamilton, Baylor Research Institute.

**Tables**

**Supplemental Table 1. Summary of Motor Level at Baseline^a^**

| Characteristic | Placebo  N=29 | | VX-210 9 mg  N=32 | |
| --- | --- | --- | --- | --- |
|  | Left | Right | Left | Right |
| Patients with motor level at baseline, n | 29 | 29 | 31 | 31 |
| Motor level, n (%) | | | | |
| C1 | 0 | 0 | 1 (3.2) | 1 (3.2) |
| C2 | 0 | 0 | 0 | 0 |
| C3 | 1 (3.4) | 1 (3.4) | 0 | 0 |
| C4 | 6 (20.7) | 6 (20.7) | 4 (12.9) | 4 (12.9) |
| C5 | 15 (51.7) | 17 (58.6) | 17 (54.8) | 16 (51.6) |
| C6 | 6 (20.7) | 4 (13.8) | 7 (22.6) | 8 (25.8) |
| C7 | 1 (3.4) | 1 (3.4) | 2 (6.5) | 2 (6.5) |

n, number of patients with non-missing assessments in each motor level at baseline; N, number of patients randomized and dosed.

^a^ The percentage for each motor level was calculated using the number of patients with motor level at baseline as the denominator.
